# The Anti-Periodontitis Effects of Ethanol Extract Prepared Using *Lactobacillus paracasei* subsp. *paracasei* NTU 101

**DOI:** 10.3390/nu10040472

**Published:** 2018-04-12

**Authors:** Te-Hua Liu, Tsung-Yu Tsai, Tzu-Ming Pan

**Affiliations:** 1 Department of Biochemical Science & Technology, National Taiwan University, Taipei 10617, Taiwan; d04b22005@ntu.edu.tw; 2 Department of Food Science, Fu Jen Catholic University, New Taipei City 24205, Taiwan; tytsai@mail.fju.edu.tw

**Keywords:** *Lactobacillus paracasei* subsp. *paracasei* NTU 101, fermented skim milk, anti-periodontitis, anti-inflammation, osteoclast differentiation

## Abstract

Poor oral health and related diseases, including caries, periodontal disease, and oral cancer, are highly prevalent across the world, particularly in the elderly. This study aimed to investigate the anti-periodontitis activity of fermented skim milk produced using the promising probiotic *Lactobacillus paracasei* subsp. *paracasei* NTU 101 (NTU101FM). An initial analysis found that an ethanol extract of NTU101FM displayed anti-oxidative activities. Further investigation of pathogen growth inhibition zones, minimum inhibitory concentrations (MICs), and minimum bactericidal concentrations (MBCs) revealed that the NTU101FM ethanol extract also had anti-periodontal pathogen activities. In addition, the NTU101FM ethanol extract significantly decreased the release of pro-inflammatory cytokines induced by lipopolysaccharide (LPS) in RAW 264.7 macrophage cells. Finally, the NTU101FM ethanol extract was found to inhibit receptor activator of nuclear factor-κB ligand (RANKL)-induced osteoclast differentiation by reducing tartrate-resistant acid phosphatase (TRAP) activity and the number of TRAP-positive multinucleated osteoclasts. In summary, our study demonstrated that ethanol extract prepared from NTU101FM has potential use as an anti-periodontitis agent.

## 1. Introduction

Oral disease is a wide-spread and global health concern that has many different causes and manifestations. In particular, periodontal disease, the major cause of tooth loss in adults, is caused by inflammatory processes that occur in gingival tissues in response to bacterial accumulation on the teeth. Chronic periodontitis is classified as slight, moderate, or severe periodontitis [1,2,3]. Recently, several studies have indicated that *Porphyromonas gingivalis* and *Aggregatibacter actinomycetemcomitans* are the main pathogens associated with periodontal disease [4,5,6]. These pathogens and associated factors, particularly the lipopolysaccharide (LPS) endotoxin found in their cell walls, are crucial mediators of the inflammatory processes that occur in gingival tissues. They induce their effects by enhancing the release of pro-inflammatory cytokines such as interleukin (IL)-1β, IL-6, and tumor necrosis factor-α (TNF-α) [7,8]. There are also several studies that indicate that oxidative stress, caused by reactive oxygen species (ROS), is an important factor that also contributes to periodontitis. Additionally, antioxidants have been found to display inhibitory effects on periodontitis and alveolar bone loss (ABL) [9,10]. Such ABL is often caused by an imbalance between bone resorption and formation. This imbalance is linked to the activity of receptor activator of nuclear factor-κB ligand (RANKL) and the release of various pro-inflammatory cytokines [11].

Further work has suggested that probiotics may offer additional scope for improving oral healthcare and have been shown to be effective for many oral diseases, including periodontitis [12]. This is likely because imbalances between probiotic and pathogenic bacteria can increase the risk of periodontal disease [12]. *Lactobacillus acidophilus*, *L. casei*, *L. fermentum*, *L. plantarum*, *L. rhamnosus*, and *L. salivarius* are all common probiotic species found in saliva. Importantly, some of these species have been shown to associate with improved outcomes in patients with periodontal disease. For example, *L. gasseri* and *L. fermentum* are both more abundant in healthy individuals than in patients with periodontal disease [13]. *L. paracasei* ssp. *paracasei* and *L. rhamnosus* have also been demonstrated to be highly antagonistic towards major oral pathogens, such as *Streptococcus mutans* and *P. gingivalis* [14]. Finally, some probiotics can ameliorate the symptoms of periodontal disease by regulating various pro- and anti-inflammatory factors that are secreted by endothelial cells. For example, Krasse et al. (2006) [15] showed that *L. reuteri* can inhibit the growth of pathogenic bacteria through the production of the bacteriocins, reuterin and reutericyclin. This allows it to compete with pathogenic bacteria for adsorption sites and inhibits the release of inflammatory factors, subsequently improving periodontal inflammation symptoms in the patient [16,17].

One potential probiotic that may have anti-pathogenic activity is *L. paracasei* subsp. *paracasei* NTU 101 (NTU 101), which was originally isolated from the feces of an aboriginal neonate in Taiwan [18]. The isolate possesses numerous biological functions; research has indicated that NTU 101 has significant antioxidant activity [19], and that fermented NTU 101 preparations exert an immunomodulatory effect, potentially by inhibiting the inflammatory response [20,21]. In addition, NTU 101 has been shown to have inhibitory effects on the growth rates of many bacteria and yeast species, with the notable exception of *Staphylococcus aureus*; both NTU 101 and NTU 101-fermented soy skim milk have been shown to exert anti-caries effects and a reduction in the incidence of tooth decay [22,23]. However, there have been few studies investigating whether similar lactic acid bacteria (LAB) fermentations have any anti-periodontitis activity.

To investigate this potential activity, we investigated the anti-periodontitis activity of NTU 101-fermented skim milk (NTU101FM). Our data strongly suggest that ethanol extracts prepared from NTU101FM have potential use as anti-periodontitis agents, which can be used to improve outcomes for patients.

## 2. Materials and Methods

### 2.1. Materials

*P. gingivalis* BCRC 14417, *A. actinomycetemcomitans* BCRC 80375, and RAW 264.7 cells were purchased from the Bioresource Collection and Research Center (BCRC, Hsinchu, Taiwan). Dimethyl sulfoxide (DMSO), 3-(4,5-dimethylthiazol-2-yl)-2,5-diphenyltetrazolium bromide (MTT), l-glutamine, LPS, and receptor activator of nuclear factor-κB ligand (RANKL) were all purchased from Sigma-Aldrich Corp. (St. Louis, MO, USA). Brain heart infusion agar, Mueller-Hinton agar, and tryptic soy broth were purchased from BD Co. (Franklin Lakes, NJ, USA). Fetal bovine serum (FBS) and Dulbecco’s modified Eagle’s medium (DMEM) were purchased from HyClone Laboratories (Logan, UT, USA). A tartrate-resistant acid phosphatase (TRAP) & alkaline phosphatase (ALP) Double-stain Kit were purchased from Takara Bio Inc. (Kusatsu, Shiga, Japan) and pro-inflammatory cytokine assay kits (IL-1β, IL-6, IL-17, and TNF-α) were purchased from BioLegend (San Diego, CA, USA).

### 2.2. Fermented Skim Milk with NTU 101 and Extraction

For the fermentation, 25% (*w*/*v*) skim milk (Anchor, Auckland, New Zealand) was warmed in a water bath at 95 °C for 1 h. The milk was then allowed to cool to 37 °C and inoculated with a 1% (*v*/*v*) suspension of NTU 101. It was then incubated at 37 °C for 2 days before being freeze-dried using a SDF-25 freeze dryer (Chang Jung Business Co., Feng-Jen, Taiwan). Material was then extracted from the freeze-dried NTU 101-fermented skim milk (NTU101FM) powders using 95% ethanol or water by shaking in a rotary shaker at 180 rpm and 37 °C for 1 h. The extracts were then centrifuged at 4 °C and 10,000× *g* for 30 min to obtain the supernatant. The supernatants were then dried in vacuo and stored at −20 °C until use.

### 2.3. Evaluation of the Anti-Oxidant Properties of NTU101FM

Several methods were used to assess the anti-oxidant properties of NTU101FM. First, the 2,2-diphenyl-1-picrylhydrazyl (DPPH)-eliminating activity was measured using 100 µL of extracts of NTU101FM added to 500 µL of 0.1 mM DPPH solution and mixed. After incubation at room temperature (approximately 25 °C) for 30 min in the dark, the absorbance at 517 nm was measured for each mixture [24]. The reducing power of NTU101FM was assessed by adding 100 µL of each extract to 100 µL of 1% K_3_Fe(CN)_6_ and 100 µL of 0.2 M phosphate-buffered saline (PBS). After incubation at 50 °C for 20 min, 500 µL 10% trichloroacetic acid (TCA) solution was added and the mixture was centrifuged at 4 °C and 3500× *g* for 10 min to obtain the supernatant. Double-distilled water and 0.1% FeCl_3_ (*w*/*v*) were added to the supernatant at a ratio of 1:1:1. The absorbance at 700 nm of each mixture was then measured [25]. Finally, the Fe^2+^-chelating activity was measured using a previously outlined method [26].

### 2.4. Anti-Microbial Activity of NTU101FM Ethanol Extract

To establish the anti-microbial activity of NTU101FM, agar diffusion assays similar to previous studies were performed, with slight modifications [27,28]. Briefly, the assays utilized 1 × 10^9^ colony-forming units (CFU)/mL of each indicator bacterial species (*P. gingivalis* and *A. actinomycetemcomitans*) that were incubated using the same volumes of agar. The surfaces of the agar plates were swabbed several times with bacteria to ensure an even distribution of each indicator species. Next, a 7-mm diameter hollow tube was used to prepare four wells on each plate, into which 50 µL of various concentrations (25 to 200 mg/mL) of NTU101FM ethanol extract were added within a 10-min period. The plates were then incubated at 37 ℃ in an anaerobic incubator for 2 or 4 days. The diameter of the inhibition zone around each well was then measured.

To establish the lowest concentration at which there was inhibition of bacterial growth, minimum inhibitory concentrations (MICs) were measured according to previous studies, with some modifications [28]. Briefly, 1 × 10^9^ CFU/mL indicator bacteria (*P. gingivalis* or *A. actinomycetemcomitans*) were incubated with different concentrations of NTU101FM ethanol extract. After incubation, the MICs were determined by visual inspection and by noting the concentrations at which there was no visible growth. Finally, the minimum bactericidal concentrations (MBCs) were measured with previously described method [29].

### 2.5. Cell Culture and Cell Viability

RAW 264.7 mouse cells were maintained in DMEM medium with 10% FBS at 37 °C, 95% humidity, and 5% CO_2_. For passage, cells were dislodged from the dish substrate using a cell scraper, aspirated, and added to new dishes when required. The culture medium was replaced every 2–3 days. To determine cell viability, RAW 264.7 cells were seeded at 1 × 10^4^ cells/well on a 24-well plate. After 24 h, the cells were incubated with NTU101FM ethanol extract for a further 24 h. Cell viability was then assessed using MTT assays and calculated using the following equation: cell viability (% of control) = (OD_sample_/OD_control_) × 100%, where OD is optical density at 595 nm.

### 2.6. Measurement of Nitric Oxide (NO) Production Levels

NO content was measured as previously described [30]. Briefly, RAW 264.7 cells were seeded at 1 × 10^4^ cells/well on a 24-well plate and left for 48 h. The cells were then incubated with NTU101FM ethanol extract for a further 24 h. To determine the NO levels, 50 µL aliquots of the cultured supernatants or standard were mixed with 50 µL sulfanilamide solution (1% sulfanilamide in 5% phosphoric acid) and 50 µL 0.1% *N*-(1-naphthyl) ethylenediamine dihydrochloride (NED) solution. The mixture was then incubated at room temperature for 10 min in the dark and the absorbance of each mixture was measured.

### 2.7. Measurement of Pro-Inflammatory Cytokine (IL-1β, IL-6, IL-17, and TNF-α) Levels

The levels of IL-1β (No. 432605), IL-6 (No. 431305), IL-17 (No. 432505), and TNF-α (No. 430905) in the cultured supernatant were tested using enzyme-linked immunosorbent assay (ELISA) kits (BioLegend, San Diego, CA, USA) according to the manufacturer-provided protocols.

### 2.8. Osteoclast Differentiation, Tartrate-Resistant Acid Phosphatase (TRAP) Staining, and TRAP Activity

To assess the effects of NTU101FM ethanol extract on osteoclast differentiation, 1 × 10^4^ cells/well RAW 264.7 cells were plated onto 24-well plates. After 24 h, the medium was substituted and the cells were co-cultured for a further 4 days in DMEM complemented with 10% FBS, 100 ng/mL RANKL, and different concentrations (25 to 500 µg/mL) of NTU101FM ethanol extract.

On the fourth day of differentiation, the cultured supernatants were removed and discarded. The cells were washed once with PBS, and 250 µL of fixation solution (citrate buffer containing 60% acetone and 10% methanol) was added. The cells were then left at room temperature for 5 min. Finally, cells were stained using a TRACP & ALP Double-stain Kit (Cat. MK300; Takara Bio Inc., Kusatsu, Shiga, Japan). Images of TRAP-positive cells were captured using a TS100 inverted microscope with a camera (Nikon, Tokyo, Japan). The TRAP activity of these cells under different treatment options were tested by ELISA.

### 2.9. Assessment of Bone Resorptive Area by Pit Formation Assay

To assess bone resorption, a pit formation assay was used with culturing methods similar to those for osteoclast differentiation, although 1 × 10^4^ RAW 264.7 cells/well were plated onto Corning Osteo Assay Surface Multiwell plates (Corning Inc., Corning, NY, USA). After 24 h, the cells were treated with RANKL (100 ng/mL) and NTU101FM ethanol extract for 4 days. The cells were then removed using 1 N NaOH for 15 min, and the resorbed areas were observed under a TS100 inverted microscope (Nikon, Tokyo, Japan) and analyzed using Image J [31].

### 2.10. Statistical Analysis

All data are represented as the mean ± standard deviation (SD) from three independent experiments. A Duncan’s multiple range test with a post hoc analysis was used for statistical testing using SPSS software (version 21, IBM Software, Armonk, NY, USA). The threshold for statistical significance was set as *p <* 0.05.

## 3. Results and Discussion

### 3.1. The Anti-Oxidative Activities of NTU101FM

Chapple and Matthews (2007) [9] previously reported that oxidative stress is a key factor that contributes to periodontitis, affecting many interactions between the host and pathogen. When the redox-state of a host is unbalanced, periodontal disease can be exacerbated. In addition, increasing the activities or abundances of anti-oxidative enzymes, or otherwise decreasing oxidative stress in the host, can improve periodontal status [10]. In this study, we assessed the anti-oxidative properties of NTU101FM using DPPH elimination, reducing power, and Fe^2+^-chelating assays. As shown in Table 1, the DPPH eliminating activities of water and ethanol NTU101FM extract at 20 mg/mL were significantly higher than that of unfermented skim milk by 14.47% and 15.40%, respectively (*p <* 0.05). These activities also demonstrated a dose-dependent effect. In addition, both unfermented and NTU101FM extracts increased the total reducing activity in a dose-dependent manner, although the ethanol extract was more effective. Finally, the Fe^2+^-chelating activity was significantly enhanced by NTU101FM (*p <* 0.05, data not shown). These results demonstrated that skim milk fermented with NTU 101 possessed anti-oxidative activities.

### 3.2. The Anti-Microbial Activities of NTU101FM

Based on the results of the anti-oxidation assays, we hypothesized that the NTU101FM ethanol extract would be more effective than the water extract. Therefore, NTU101FM ethanol extract was used for subsequent assays to assess the inhibitory ability of NTU 101 on periodontal pathogens. Several factors have been found to promote periodontal disease, including eating habits, poor oral hygiene, and faulty dental restorations. However, the main factor is dental plaque formed by various oral pathogens [32,33]. Therefore, inhibiting the growth of periodontal pathogens would effectively prevent or improve the periodontal status. The degree of growth inhibition against *P. gingivalis* and *A. actinomycetemcomitans* by NTU101FM ethanol extract are shown in Table 2. These results showed that both *P. gingivalis* and *A. actinomycetemcomitans* were inhibited by treatment with 200 mg/mL NTU101FM ethanol extract. The diameters of the growth inhibition zones for *P. gingivalis* and *A. actinomycetemcomitans* were 16.50 ± 0.20 and 22.75 ± 0.35 mm, respectively. In particular, treatment with 25 to 200 mg/mL of NTU101FM ethanol extract still led to a noticeable inhibition zone in *A. actinomycetemcomitans*. MIC and MBC comparisons are shown in Table 3. The MICs and MBCs of the NTU101FM ethanol extract were both 30 mg/mL for *P. gingivalis*, and were 1.0 and 2.5 mg/mL, respectively, for *A. actinomycetemcomitans*.

Several prior studies have reported that certain *Lactobacillus* spp. can produce bacteriocins. These can often feature better anti-microbial effects than traditional antibiotics [34]. Furthermore, Sookkhee et al. (2001) [14] found that enhancing the abundance of oral *L. paracasei* ssp. *paracasei* (D6, D4, N14) or *L. rhamnosus* could decrease the number of periodontal pathogens. In addition, bacteriocins produced by *L. paracasei* HL32 can destroy the cytomembranes of *P. gingivalis*, decreasing its abundance [35]. Previously, NTU 101 has been shown to possess anti-microbial activity against a wide variety of pathogens, including Gram-negative bacteria [22]. We therefore concluded that our NTU101FM ethanol extract contained the specific anti-microbial ingredient(s) that acts against periodontal pathogens.

### 3.3. RAW 264.7 Cell Viability after Treatment with NTU101FM Ethanol Extract

**Figure 1:**
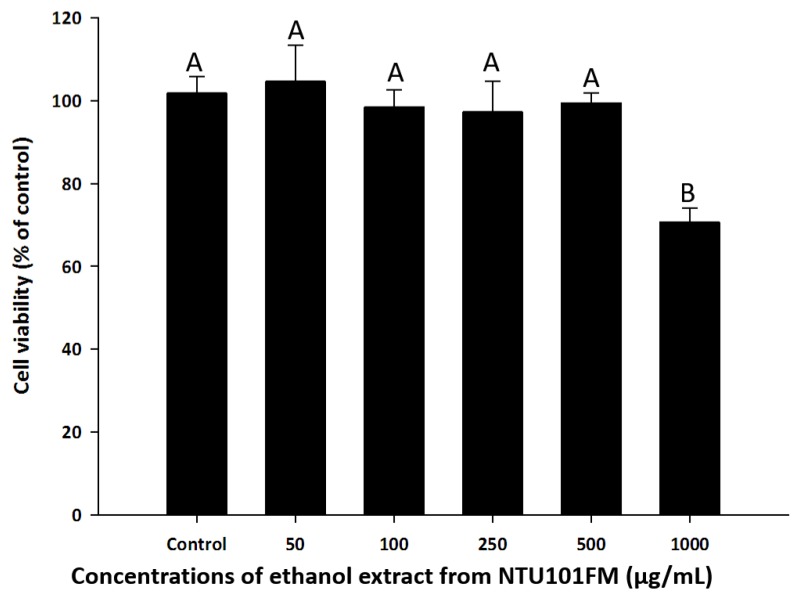
Effects of NTU101FM ethanol extract on cell viability of RAW 264.7 cell. Cells were treated with various concentrations of NTU101FM ethanol extract (50 to 1000 µg/mL) for 24 h. Cell viability was measured by MTT assay and represented as percent of control cell viability. The data are presented as means ± SD (*n* = 3). Values with different uppercase letters were significant by Duncan’s multiple range tests (*p <* 0.05). NTU101FM, NTU 101-fermented skim milk; MTT, 3-(4,5-dimethylthiazol-2-yl)-2,5-diphenyltetrazolium bromide.

To find a safe yet effective concentration of NTU101FM ethanol extract, we measured the cell viability of RAW 264.7 cells after treatment with various concentrations of the extract. As shown in Figure 1, there was no cytotoxicity observed in RAW 264.7 cells between 50 and 500 µg/mL NTU101FM ethanol extract. We therefore decided to use concentrations below 500 mg/mL in subsequent experiments.

### 3.4. The Anti-Inflammatory Activities of NTU101FM Ethanol Extract on RAW 264.7 Cells

**Figure 2:**
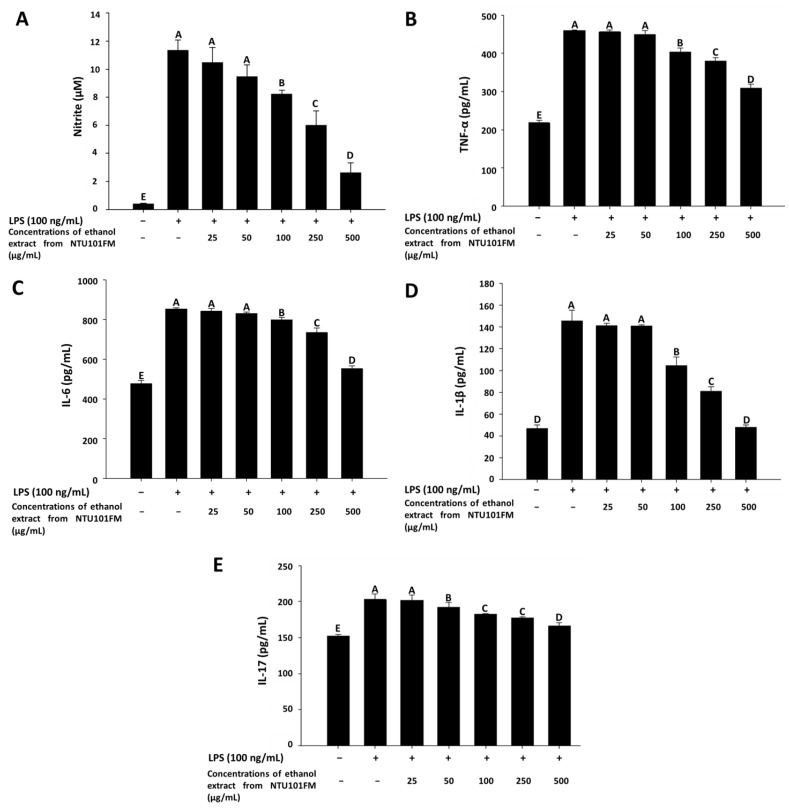
Effects of NTU101FM ethanol extract on: (**A**) NO; (**B**) TNF-α; (**C**) IL-6; (**D**) IL-1β; and (**E**) IL-17 of LPS-induced inflammation RAW 264.7 cell. Cells were treated with various concentrations of NTU101FM ethanol extract (25 to 500 µg/mL) for 24 h. The data are presented as means ± SD (*n* = 3). Values with different uppercase letters were significant by Duncan’s multiple range tests (*p* < 0.05). NTU101FM, NTU 101-fermented skim milk; NO, nitric oxide; TNF-α, tumor necrosis factor-α; IL-6, interleukin-6; IL-1β, interleukin-1β; IL-17, interleukin-17; LPS, lipopolysaccharide.

As previously described, periodontitis is a chronic inflammatory disease. In mediating their probiotic effects against periodontitis, *Lactobacillus* spp. may either regulate the immune response or reduce inflammation [36]. Even though the anti-inflammatory mechanisms remain unclear, Cotter et al. (2005) [37] showed that these effects may associate with polysaccharides, peptidoglycans, or bacteriocins secreted by *Lactobacillus* spp. In addition, when the levels of NO are too high, the peroxidation of free radicals can lead to negative cytotoxicity or inflammatory reactions [38]. However, NO is not typically produced by non-activated macrophages and is only secreted after activation by certain cytokines (such as IL-1 and TNF-α) or LPS [39]. Therefore, LPS-induced inflammation in a RAW 264.7 cell model can be used to assess the levels of inflammation and the impact of treatments on inflammatory processes. Using this LPS-induced inflammation model, we measured the effects of NTU101FM ethanol extract on NO, IL-1β, IL-6, TNF-α, and IL-17 levels. We initially confirmed that RAW 264.7 cells do not produce pro-inflammatory cytokines after treatment with 25 to 500 mg/mL of NTU101FM ethanol extract when compared to the control group (data not shown). After RAW 264.7 cells were co-cultured with 100 ng/mL LPS for 24 h, NO production significantly increased 27.51-fold relative to control group (*p <* 0.05; Figure 2A). However, treatment with 100 to 500 µg/mL NTU101FM ethanol extract successfully reduced NO levels in the LPS-induced RAW 264.7 macrophage model (Figure 2A). In addition, the levels of TNF-α, IL-6, IL-1β, and IL-17 in the cellular supernatants of the LPS groups also significantly increased to 460.45 ± 0.56 pg/mL (111.18%), 854.25 ± 5.96 pg/mL (78.66%), 145.50 ± 9.82 pg/mL (210.68%), and 202.96 ± 7.70 pg/mL (33.44%), respectively (*p <* 0.05; Figure 2B–E). Treatment with NTU101FM ethanol extract (100 to 500 μg/mL) significantly reduced the levels of TNF-α (by 14.15% to 48.76%), IL-6 (by 6.87% to 54.53%), IL-1β (by 39.12% to 204.18%), and IL-17 (by 5.60% to 22.12%), when compared to the LPS-only groups (*p <* 0.05). These results indicated that treatment with NTU101FM ethanol extract regulates the anti-inflammatory responses by reducing the levels of pro-inflammatory cytokines induced by LPS.

### 3.5. The Inhibitory Effects of NTU101FM Ethanol Extract on RANKL-Induced Osteoclastogenesis

**Figure 3:**
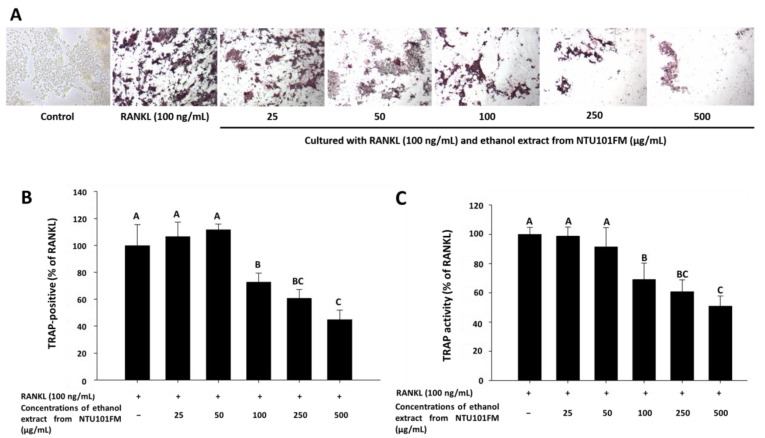
Effect of NTU101FM ethanol extract on RANKL-induced osteoclast differentiation: (**A**) photograph of TRAP-stained osteoclasts; (**B**) number of TRAP-positive multinucleated osteoclasts; and (**C**) TRAP activity. The RAW 264.7 cells were cultured with the indicated dose of NTU101FM ethanol extract (25 to 500 μg/mL) in the presence of RANKL (100 ng/mL) for four days. The data are presented as means ± SD (*n* = 3). Values with different uppercase letters were significant by Duncan’s multiple range tests (*p* < 0.05). NTU101FM, NTU 101-fermented skim milk; RANKL, receptor activator of nuclear factor kappa-β ligand; TRAP, tartrate-resistant acid phosphatase.

There is increasing evidence that alveolar bone loss (ABL) is an important indicator of periodontitis [40]. ABL depends on a balance between bone resorption and bone formation. Osteoclasts are large, multinucleated cells that originate from a macrophage lineage and induce bone resorption. This process is stimulated by RANKL and is induced into an overactive state by certain inflammatory cytokines, such as TNF-α, IL-1, and IL-6 [41,42,43]. Previously, Ciucci et al. (2015) [44] reported that IL-17 and TNF-α were also regulators of osteoclastogenesis, either directly or indirectly. RANKL is an important protein that regulates osteoclast differentiation and function. Inhibiting osteoclastogenesis by reducing inflammation and osteoclast differentiation via RANKL is considered a possible strategy for preventing and ameliorating periodontitis [43]. To address whether NTU 101 could affect osteoclastogenesis, we first examined osteoclast differentiation using TRAP staining (Figure 3A). This showed that NTU101FM ethanol extract from concentrations of 100 to 500 µg/mL could suppress RANKL-induced osteoclast differentiation in RAW 264.7 cells. We also quantified the number of TRAP-positive multinucleated osteoclasts (Figure 3B), finding that the number of TRAP-positive multinucleated osteoclasts decreased after treatment with NTU101FM ethanol extract from 100 to 500 µg/mL (*p <* 0.05). Compared to the RANKL-induced group treated only with 100 ng/mL RANKL, the NTU101FM ethanol extract significantly reduced the number of TRAP-positive multinucleated osteoclasts by 27.22% to 55.13% (*p <* 0.05). We next assessed TRAP activity in RAW 264.7 cells cultured with various doses of NTU101FM ethanol extract in the presence of RANKL for four days (Figure 3C). This revealed that the TRAP activities of differentiated RAW 264.7 cells were similar to the number of TRAP-positive multinucleated osteoclasts. However, NTU101FM ethanol extract (100 to 500 µg/mL) decreased TRAP activity by 30.99% to 49.07% (*p <* 0.05). These findings suggest that NTU101FM ethanol extract may reduce bone resorption by inhibiting the RANKL-induced osteoclast differentiation.

### 3.6. The Inhibitory Effects of NTU101FM Ethanol Extract on Bone Resorption

**Figure 4:**
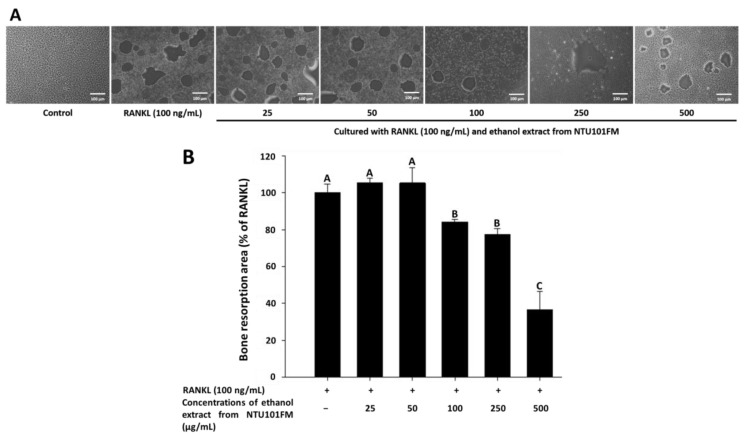
Effect of NTU101FM ethanol extract on RANKL-induced bone resorption: (**A**) photograph; and (**B**) the area in osteoclast. The RAW 264.7 cells were cultured with the indicated dose of NTU101FM ethanol extract (25 to 500 μg/mL) in the presence of RANKL (100 ng/mL) for four days. The data are presented as means ± SD (*n* = 3). Values with different uppercase letters were significant by Duncan’s multiple range tests (*p* < 0.05). NTU101FM, NTU 101-fermented skim milk; RANKL, receptor activator of nuclear factor kappa-β ligand.

Finally, we used a Corning Osteo Assay Surface system to measure the extent of bone resorption stimulated by RANKL (representative images are shown in Figure 4A). We observed a significant increase in the areas of bone resorption when RAW 264.7 cells were induced by RANKL for four days. However, after treatment with 100, 250, and 500 μg/mL NTU101FM ethanol extract, these areas of bone resorption were reduced by 15.85%, 22.67%, and 63.30%, respectively (*p <* 0.05; Figure 4B). This suggests that NTU101FM ethanol extract has an inhibitory effect on RANKL-induced bone resorption.

## 4. Conclusions

**Figure 5:**
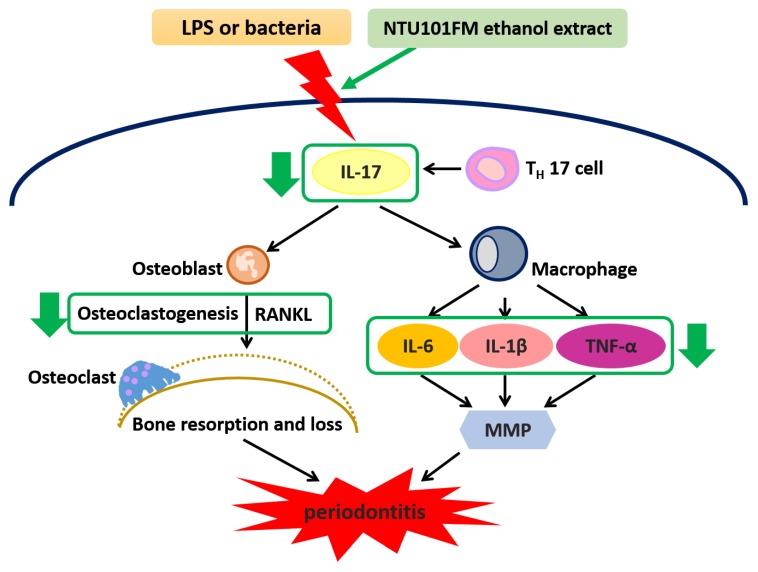
The proposed mechanism of NTU101FM ethanol extract to attenuate periodontal disease and its associated symptoms. LPS, lipopolysaccharide; IL-17, interleukin 17; T_H_ 17 cell, T helper type 17 cell; RANKL, receptor activator of nuclear factor kappa-B ligand; IL-6, interleukin 6; IL-8, interleukin 8; MMP, matrix metallopeptidase; IL-1β, interleukin 1β; TNF-α, tumor necrosis factor-α.

Our data have revealed that an NTU101FM ethanol extract displayed significant anti-oxidative activities when assessed by DPPH elimination, reducing power, and Fe^2+^-chelating assays. Furthermore, NTU101FM ethanol extract could decrease the growth of both *P. gingivalis* and *A. actinomycetemcomitans*, pathogens majorly associated with periodontitis. Our study also demonstrated that NTU101FM ethanol extract affected LPS-induced inflammatory responses and RANKL-induced osteoclast differentiation in RAW 264.7 cells. Finally, we have demonstrated that NTU101FM ethanol extract inhibited RANKL-induced osteoclast differentiation by reduced TRAP activity and the total number of TRAP-positive multinucleated osteoclasts. NTU101FM ethanol extract also decreased the levels of RANKL-induced bone resorption. A summary of the proposed mechanism is shown in Figure 5. Our study is the first study to examine the potential role of an NTU101FM ethanol extract in combating periodontal disease. Our results strongly suggest that such an extract has the potential to act as a therapeutic agent for the prevention and treatment of periodontitis. However, the study was limited in that it used an in vitro model. Experiments using an LPS-induced periodontal disease animal model are currently being performed. It will confirm if our conclusions can be replicated in vivo. Such confirmation would suggest that an ethanol extract prepared using NTU 101 fermentation would have therapeutic applications that may improve the outcomes for patients with periodontal disease.

## Figures and Tables

**Table 1:** The 1,1-diphenyl-2-picrylhydrazyl (DPPH) eliminating and reducing power activities of unfermented skim milk or NTU101FM extracts.

Concentration (mg/mL)	DPPH Eliminating Effect (%)				Reducing Power (OD_700_)			
	UF EE	UF WE	NTU 101 EE	NTU 101 WE	UF EE	UF WE	NTU 101 EE	NTU 101 WE
20.0	50.94 ± 2.06 ^Ac^	45.11 ± 4.24 ^Ac^	66.34 ± 3.57 ^Aa^	59.58 ± 4.17 ^Ab^	0.451 ± 0.03 ^Ac^	0.215 ± 0.01 ^Ad^	0.604 ± 0.02 ^Aa^	0.350 ± 0.01 ^Ab^
10.0	42.55 ± 1.51 ^Bc^	37.36 ± 1.03 ^Bd^	59.66 ± 1.64 ^Ba^	54.15 ± 1.55 ^Bb^	0.389 ± 0.00 ^Bb^	0.207 ± 0.00 ^ABd^	0.507 ± 0.02 ^Ba^	0.288 ± 0.01 ^Bc^
5.0	35.58 ± 0.57 ^Cc^	20.37 ± 0.92 ^Cd^	54.67 ± 1.65 ^Ca^	41.26 ± 1.70 ^Cb^	0.343 ± 0.00 ^Cb^	0.187 ± 0.00 ^Bd^	0.381 ± 0.00 ^Ca^	0.261 ± 0.01 ^BCc^
1.0	27.11 ± 2.49 ^Dc^	N.D.	45.36 ± 1.98 ^Da^	35.24 ± 1.01 ^Db^	0.276 ± 0.00 ^Da^	0.121 ± 0.01 ^Cb^	0.275 ± 0.01 ^Da^	0.251 ± 0.04 ^CDa^
0.1	16.06 ± 1.54 ^Ec^	N.D.	40.95 ± 2.09 ^Ea^	28.11 ± 2.21 ^Eb^	0.207 ± 0.00 ^Eb^	0.070 ± 0.02 ^Dc^	0.236 ± 0.01 ^Ea^	0.225 ± 0.01 ^Dab^

Data are presented as mean ± SD (*n* = 3). Values with different uppercase letters were significantly different in column and values with different lowercase letters were significantly different in row (*p <* 0.05). DPPH: 0.1 mM. UF EE: ethanol extract of unfermented skim milk; UF WE: water extract of unfermented skim milk; NTU 101 EE: ethanol extract of *L. paracasei* subsp. *paracasei* NTU 101-fermented skim milk; NTU 101 WE: water extract of *L. paracasei* subsp. *paracasei* NTU 101-fermented skim milk; N.D.: non-detected.

**Table 2:** Inhibition zone of NTU101FM ethanol extract against periodontal pathogens.

Concentrations (mg/mL)	Inhibition Zone (Diameter, mm) ^a,b^	
	*P. gingivalis* BCRC 14417	*A. actinomycetemcomitans* BCRC 14405
200	16.50 ± 0.20	22.75 ± 0.35
100	-	19.50 ± 1.41
50	-	18.50 ± 0.71
25	-	11.75 ± 1.06

^a^ Inhibition zone does not include the disc diameter (7 mm). ^b^ - No inhibition zone. The cultures, containing approximately 1 × 10^9^ colony-forming unit (CFU)/mL of indicator bacteria, were inoculated on the agar plate. NTU101FM, NTU 101-fermented skim milk.

**Table 3:** Minimum inhibitory concentration (MIC) and minimum bactericidal concentration (MBC) of NTU101FM ethanol extract against periodontal pathogens.

Periodontal Pathogens	MIC (mg/mL)	MBC (mg/mL)
*P. gingivalis* BCRC 14417	30.00	30.00
*A. actinomycetemcomitans* BCRC 14405	1.00	2.50

## Abbreviations

ABL	alveolar bone loss
ALP	alkaline phosphatase
DMEM	Dulbecco’s modified Eagle’s medium
DMSO	dimethyl sulfoxide
DPPH	2,2-diphenyl-1-picrylhydrazyl
ELISA	enzyme-linked immunosorbent assay
FBS	fetal bovine serum
IL	interleukin
LAB	lactic acid bacteria
LPS	lipopolysaccharide
MBCs	minimum bactericidal concentrations
MICs	minimum inhibitory concentrations
MTT	3-(4,5-dimethylthiazol)-2-yl-2,5-diphenyltetrazolium bromide
NO	nitric oxide
NTU 101	*Lactobacillus paracasei* subsp. *paracasei* NTU 101
NTU101FM	NTU 101-fermented skim milk
PBS	phosphate-buffered saline
RANKL	receptor activator of nuclear factor-κB ligand
ROS	reactive oxygen species
TCA	trichloroacetic acid
TNF	tumor necrosis factor
TRAP	tartrate-resistant acid phosphatase
